# Protein Function Prediction Based on PPI Networks: Network Reconstruction vs Edge Enrichment

**DOI:** 10.3389/fgene.2021.758131

**Published:** 2021-12-14

**Authors:** Jiaogen Zhou, Wei Xiong, Yang Wang, Jihong Guan

**Affiliations:** ^1^ Jiangsu Provincial Engineering Research Center for Intelligent Monitoring and Ecological Management of Pond and Reservoir Water Environment, Huaiyin Normal University, Huian, China; ^2^ Shanghai Key Lab of Intelligent Information Processing, and School of Computer Science, Fudan University, Shanghai, China; ^3^ Department of Computer Science and Technology, Tongji University, Shanghai, China

**Keywords:** edge enrichment, network reconstruction, protein-protein interaction networks, protein function prediction, protein sequence annotation

## Abstract

Over the past decades, massive amounts of protein-protein interaction (PPI) data have been accumulated due to the advancement of high-throughput technologies, and but data quality issues (noise or incompleteness) of PPI have been still affecting protein function prediction accuracy based on PPI networks. Although two main strategies of *network reconstruction* and *edge enrichment* have been reported on the effectiveness of boosting the prediction performance in numerous literature studies, there still lack comparative studies of the performance differences between *network reconstruction* and *edge enrichment*. Inspired by the question, this study first uses three protein similarity metrics (local, global and sequence) for network reconstruction and edge enrichment in PPI networks, and then evaluates the performance differences of network reconstruction, edge enrichment and the original networks on two real PPI datasets. The experimental results demonstrate that edge enrichment work better than both network reconstruction and original networks. Moreover, for the edge enrichment of PPI networks, the sequence similarity outperformes both local and global similarity. In summary, our study can help biologists select suitable pre-processing schemes and achieve better protein function prediction for PPI networks.

## 1 Introduction

Over the past decades, massive amounts of un-annotated protein sequence data have been accumulated with the advancement of high-throughput biological technologies. Due to high costs and time-consummation of experimental determining protein function annotation, the proportion of annotated proteins has been still relatively low (Sharan et al., 2007; Barrell et al., 2009). The increasing efforts have been made to predict protein functions.

As the best-known and early method of protein function prediction, homology-based prediction method indeed gave rise to a series of protein function prediction methods based on protein sequence or structural similarity (Sleator and Walsh, 2010). At the same time, the emerging of available protein databases, such as FATCAT (Ye and Godzik, 2004), PAST (Täubig et al., 2006) and PROCAT (Wallace et al., 1996), has further helped to improve the effectiveness of protein prediction. However, the low sequence similarity scores often occur when comparing target protein sequences with source protein sequences (Ofran et al., 2005), and thus this significantly reduces the effective application of homology-based prediction methods.

With the increasing amounts of the measured protein-protein interaction (PPI) data, more and more protein function prediction methods based on PPI networks are proposed and generally outperform the above homology-based prediction methods. In PPI networks, proteins and protein-protein interactions are represented by nodes and edges, respectively (Sharan et al., 2007; Chen et al., 2020; Wu et al., 2020; Waiho et al., 2021). Up to now, numerous algorithms have been used in protein function prediction based on PPI networks, such as edge-betweenness clustering (Dunn et al., 2005), Graphlet-based edge clustering (Solava et al., 2012), clique percolation (Adamcsek et al., 2006), GRAAL (Kuchaiev et al., 2010), hybrid-property based method (Hu et al., 2011), and IsoRank (Singh et al., 2008). Moreover, advanced machine learning and deep learning techniques have also been used for protein function prediction, including collective classification (Xiong et al., 2013; Wu et al., 2014), active learning (Xiong et al., 2014), DeepInteract (Sunil et al., 2017), ConvsPPIS (Zhu et al., 2020), PhosIDN (Yang et al., 2021) and WinBinVec (Abdollahi et al., 2021), etc.

The above methods mainly use existing PPI data. However, current PPI data mainly generated by high-throughput or TAP-MS techniques (Berggard et al., 2007), are often in presence of noise and incompleteness, and this unavoidably causes adverse effects on the prediction performance. Two main methods of *network reconstruction* and *edge enrichment* are proposed to effectively boost the prediction performance. Different strategies are used for network reconstruction or edge enrichment. For example, Bogdanov and Singh (2010) presented a network reconstruction approach by extracting functional neighborhood features using random walk with restart. Chua et al. (2007) used weighting strategies to enrich PPI networks, and adopted a local prediction method to predict the functions of un-annotated proteins. Xiong et al. (2013) applied collective classification to PPI networks with enriched edges to predict protein functions.

Although the above two types of approaches achieve promising performance improvements, there still lack comparative studies of the performance differences between network reconstruction and edge enrichment. We do not still know which one is better in performance, or specifically, which one should be applied for different situations. Inspired by the question, we conducte a comprehensive comparison of two network transformation of network reconstruction and edge enrichment for boosting the performance of PPI network-based protein functional annotation. Concretely, we first use three different protein similarity metrics for network reconstruction and edge enrichment of PPI networks, and then evaluate the performance differences between the two transformed networks (network reconstruction and edge enrichment) and original networks on two real PPI datasets. The results of experiments demonstrate that edge enrichment work better than both network reconstruction and original networks. Moreover, for the edge enrichment of PPI networks, the sequence similarity outperformes both local and global similarity. More detailed work will be presented in later sections.

## 2 Materials and Methods

### 2.1 Similarity Metrics

As we point out above, the noise and incompleteness of PPI network data adversely affects the performance of protein functional annotation. Network reconstruction and edge enrichment are major approaches to improve PPI data quality. In this work, we carry out comparison study on these two approaches by reconstructing and enriching original networks using various protein similarity metrics, including sequence similarity, local similarity and global similarity. In what follows, we describe and discuss these similarity measures in detail.

#### 2.1.1 Protein Sequence Similarity

BLAST method (Altschul et al., 1997) is used to measure the similarity between any two proteins in this study. The similarity of a given protein *V*_*x*_ with other proteins is defined as

$$S\left(V_{x}\right)=\left[S_{x,1},S_{x,2},\ldots ,S_{x,i},\ldots ,S_{x,n}\right]$$ (1)

where *S*_*x*,*i*_ is the similarity score between the pair of proteins *V*_*x*_ and *V*_*i*_. Due to ignoring self-similarity, *S*_*x*,*i*_ = 0 is set when *x* = *i*.

#### 2.1.2 Local Similarity Indices

We consider three kinds of local similarity indices, including *Common Neighbors* (CN), *Jaccard Index* and Functional Similarity (FS).

*Common Neighbors*. Given nodes *u* and *v*, their neighboring sets are *N*_*u*_ and *N*_*v*_, respectively. The CN is defined as the neighborhood overlap of the nodes (Newman, 2001). The more identical neighbors two nodes have, the higher the CN value is. The measure of CN is as follows:

$$S_{CN}\left(u,v\right)=\left|N_{u}\cap N_{v}\right|$$ (2)

*Jaccard Index*. Given nodes *u* and *v* and their corresponding neighboring sets of *N*_*u*_ and *N*_*v*_, Jaccard index is used to measure the similarity between the *N*_*u*_ and *N*_*v*_ sets, and it is calculated as:

$$S_{Jaccard}\left(u,v\right)=\frac{\left|N_{u}\cap N_{v}\right|}{\left|N_{u}\cup N_{v}\right|}$$ (3)

*Functional Similarity* (FS). For a PPI network, FS index was first used to measure the similarity of any pair of proteins (Chua et al., 2006), and it is defined as follows:

$$S_{FS}\left(u,v\right)=\frac{2\left|N_{u}\cap N_{v}\right|}{\left|N_{u}-N_{v}\right|+2\left|N_{u}\cap N_{v}\right|+\lambda_{u,v}}\times \frac{2\left|N_{u}\cap N_{v}\right|}{\left|N_{v}-N_{u}\right|+2\left|N_{u}\cap N_{v}\right|+\lambda_{v,u}}$$ (4)

where $\lambda_{u,v}=\max\left.\left(0,n_{avg}-\left(\left|N_{u}-N_{v}\right|\right)+\left|N_{u}\cap N_{v}\right|\right)\right)$ , and by using the *λ*_*u*,*v*_ factor, similarity weights between protein pairs are penalized when their common neighbors are too few. *n*_*avg*_ is the average number of close neighbors that each node has in the network. In a weighted PPI network, the labeled weights of edges mean interaction confidences between pairs of proteins. Thus, we can modify the FS index to take into account the confidence of each interaction. The extended FS index for weighted PPI networks, named FS.R, is defined as follows:

$$\begin{array}{c} S_{FS.R}\left(u,v\right)=\frac{2\sum_{w\in \left(N_{u}\cap N_{v}\right)}r_{u,w}r_{v,w}}{\sum_{w\in N_{u}}r_{u,w}+\sum_{w\in \left(N_{u}\cap N_{v}\right)}r_{u,w}r_{v,w}+\lambda_{u,v}}\times \\ \frac{2\sum_{w\in \left(N_{u}\cap N_{v}\right)}r_{u,w}r_{v,w}}{\sum_{w\in N_{v}}r_{v,w}+\sum_{w\in \left(N_{u}\cap N_{v}\right)}r_{u,w}r_{v,w}+\lambda_{v,u}}. \end{array}$$ (5)

#### 2.1.3 Global Similarity Indices

Two global similarity indices are considered in this paper, they are Katz index and random walk with restart.

*Katz Index*. This index is proposed by Lü and Zhou (2011). It sums the set of paths directly and deals with the paths by length so that the shorter paths get more weights. Formally,

$$S_{Katz}\left(u,v\right)=\overset{\infty }{\underset{L=1}{\sum }}\beta^{L}\cdot \left|paths_{uv}^{<L>}\right|=\beta A_{uv}+\beta^{2}{\left(A^{2}\right)}_{uv}+\beta^{3}{\left(A^{3}\right)}_{uv}+\ldots$$ (6)

where $paths_{uv}^{<L>}$ is the set of the paths, which connect the nodes of *u* and *v* with a path length of L. The parameter of *β* controls the path weights.

*Random Walk with Restart (RWR)*. Tong et al. (2008) used RWR index to measure the relevance score between node *j* and node *i* in a PPI network. Given the adjacency matrix *W*_*n*,*n*_ of a PPI network, a random walker transmits from the starting node *i* to one of its neighbors at random with probability c, and returns to the node *i* with the probability 1 − *c*. Finally, the walker will stay stably at node *j* with probability *R*_*i*,*j*_. The steady-state probability *R*_*i*,*j*_ is defined as RWR index. We have

$$\vec{R_{i}}=c{\tilde{W}}^{T}\vec{R_{i}}+\left(1-c\right)\vec{e_{i}}$$ (7)

where $\vec{e_{i}}$ is the starting vector, the *i*th element is 1 and the other elements are 0. $\tilde{W}$ is a weighted matrix. For an unweighted network, ${\tilde{W}}_{ij}=1/m$ (where *m* is the number of neighbors that node *i* has) if *i* and *j* are connected, and ${\tilde{W}}_{ij}=0$ otherwise. For a weighted network,

$$\left\{\begin{array}{c} {\tilde{W}}_{ij}=W_{ij}/\overset{n}{\underset{j=1}{\sum }}W_{ij},\;\;\;if\;i\;and\;j\;are\;connected.\;\\ {\tilde{W}}_{ij}=0,\;\;\;otherwise.\; \end{array}\right.$$ (8)

#### 2.2 Network Reconstruction and Edge Enrichment

*Network reconstruction* is carried out as follows: First, the similarity scores between protein pairs in the original PPI network are calculated according to the above similarity indexes. Next, some interactions are selected to reconstruct the PPI network based on the similarity scores. As in Liben-Nowell and Kleinberg (2007), an appropriate score threshold is used such that the number of protein pairs with higher scores than the threshold is as same as possible to the interaction number of the original network. Then, a new network is formed by using the protein pairs with higher scores over the threshold. However, this approach may lead to absence of some proteins in the new network. Alternatively, for any node *N*_*i*_ in the original network, we first remove all its interactions. We find the top *k* neighbors most similar to the node *N*_*i*_. Then, the *k* edges from the node *N*_*i*_ to its top *k* neighbors are created, and their similarity scores are used as edge weights in the new network. Thus, we have

$$S{\left(N_{i}\right)}_{k}=\left[S_{i,1},S_{i,2},\ldots ,S_{i,k}\right].$$ (9)

*Edge enrichment* is also performed in two steps as in network reconstruction, the only difference is that all interactions in the original network are preserved. An enriched network has two types of edges: *explicit edges* (old edges) and similarity-inferred edges (new edges). Here, there are two questions to be addressed: One is how to combine the edge weights with different semantics, and another is how many edges are added for each protein, that is, how to optimize the parameter *k* (see Eq. 9). The questions will be discussed in the following sections.

### 2.3 Protein Function Prediction Approaches

In this study, protein function predictions on two real PPI datasets are performed using two different approaches.The first one is majority method, which is a local neighbor counting approach (Schwikowski et al., 2000). The second is a global protein function prediction approach, which is common called *collective classification* (Xiong et al., 2013). Details of this approach are presented in the following subsections.

**ALGORITHM 1:** Gibbs sampling

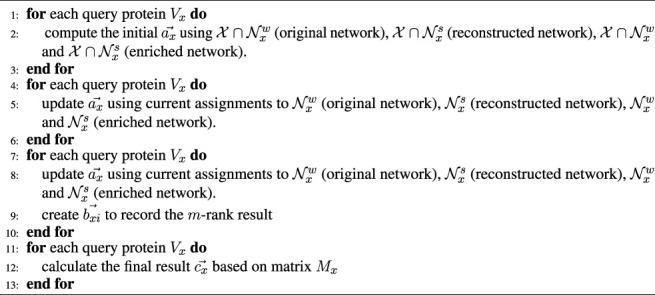

### 2.4 Gibbs Sampling Based Collective Classification

Gibbs sampling (GS) includes two main processes of *bootstrapping* and *iterative classification* (Sen et al., 2008). The pseudo-code is illustrated below.

#### 2.4.1 Bootstrapping

The closer the proteins to each other, the more similar their functions become in a PPI network. For an unannotated protein, its probability distribution is estimated using a weighted voting method. In the original or reconstructed network, there is only one kind of annotated neighbors to vote. An unannotated protein *V*_*x*_ has the corresponding explicit neighbors of *N*_*x*_ or *k* similarity-inferred neighbors. For the above neighbor sets, we have their edge weights as follows:

$$N_{x}^{w}=\left[w_{x1},w_{x2},\ldots ,w_{xi},\ldots ,w_{xN_{x}}\right]\;\;\;N_{x}^{s}=\left[S_{x,1},S_{x,2},\ldots ,S_{x,i},\ldots ,S_{x,k}\right]$$ (10)

The probability of *V*_*x*_ having the *j*th function *F*_*j*_ (*V*_*x*_ *F*_*j*_) is calculated as follows:

$$P_{x}^{j}=\frac{1}{Z_{x}^{w}}\overset{N_{x}}{\underset{i=1}{\sum }}w_{x,i}f_{i,j}\;\;\;P_{x}^{j}=\frac{1}{Z_{x}^{s}}\overset{k}{\underset{i=1}{\sum }}S_{x,i}f_{i,j}$$ (11)

where $Z_{x}^{w}$ and $Z_{x}^{s}$ are the normalizers:

$$Z_{x}^{w}=\overset{m}{\underset{j=1}{\sum }}\overset{N_{x}}{\underset{i=1}{\sum }}w_{x,i}f_{i,j}\;\;\;Z_{x}^{s}=\overset{m}{\underset{j=1}{\sum }}\overset{k}{\underset{i=1}{\sum }}S_{x,i}f_{i,j}$$ (12)

However, in the enriched network, there are both old (explicit) and new (similarity-inferred) neighbors which need to be voted. So, the parameter *λ* ∈ (0, 1) is used to combine the two types of different neighbors. Given a query protein *V*_*x*_, the *V*_*x*_ *F*_*j*_ probability is calculated as follows:

$$P_{x}^{j}=\lambda \frac{1}{Z_{x}^{w}}\overset{N_{x}}{\underset{i=1}{\sum }}w_{x,i}f_{i,j}+\left(1-\lambda \right)\frac{1}{Z_{x}^{s}}\overset{k}{\underset{i=1}{\sum }}S_{x,i}f_{i,j}$$ (13)

A higher $P_{x}^{j}$ value indicates a higher probability that protein *V*_*x*_ is more likely to have *j*th function *F*_*j*_. The *V*_*x*_ *F*_*j*_ probability distribution is represented as:

$$\vec{a_{x}}=\left[P_{x}^{1},P_{x}^{2},\ldots ,P_{x}^{m}\right]$$ (14)

### 2.4.2 Iterative Classification

Iterative classification has two main steps of burn-in and sampling. In burn-in period, iteration number is fixed, and $\vec{a_{x}}$ is updated in each iteration. In sampling period, we update $\vec{a_{x}}$ in each iteration, and also count how many times the *j*th function *F*_*j*_ for protein *V*_*x*_ are sampled. Considering each protein with one or more functions, therefore, we define the most likely function of the protein *V*_*x*_ as follow:

$$b_{x}^{j}=argmax_{j\in \left[1,m\right]}P_{x}^{j}$$ (15)

where $b_{x}^{j}$ represents the *j*th most likely function of the protein *V*_*x*_, that is the jth-rank result. We further use $\vec{b_{xi}}$ vector to record all ranking results in the *i*th iteration.

$$\vec{b_{xi}}=\left[b_{xi}^{1},b_{xi}^{2},\ldots ,b_{xi}^{m}\right].$$ (16)

The matrix *M*_*x*_ with *s* rows and *m* columns is produced after running the predetermined *s* number of iterations.

$$M_{x}={\left[\vec{b_{x1}},\vec{b_{x2}},\ldots ,\vec{b_{xs}}\right]}^{T}.$$ (17)

Finally, we obtain the required m-dimensional vector $\vec{c_{x}}$ for query protein *V*_*x*_:

$$\vec{c_{x}}=\left[c_{x}^{1},c_{x}^{2},\ldots ,c_{x}^{m}\right].$$ (18)

where $c_{x}^{1}$ is the first ranked prediction in the *i*th column of *M*_*x*_.

## 3 Results and Discussion

### 3.1 Data Preprocessing and Experimental Workflow

The two PPI datasets of A and B are used in our study. The datasets A and B are downloaded from the databases of BioGRID (Stark et al., 2011) and STRING (Szklarczyk et al., 2011), respectively. The datasets A and B are annotated as in Ashburner et al. (2000). The datasets in this study are based on Gene Ontology (GO) annotation. GO annotations consist of three basic namespaces: molecular function, biological process and cellular component. We construct one protein interaction network for each GO namespace using only physical interactions.Therefore, there are totally six PPI networks (three for *S.cerevisiae* and the other three for *M.musculus*) in Dataset A. For Dataset B, we construct two PPI networks (one for *S.cerevisiae* and another for *M.musculus*).More detailed information was listed in the supplementary material (Supplementary Table S1).

The comparison of the function prediction performance on the reconstructed and enriched networks with that on the original networks is first performed using the cross validation of leave-one-out method (LOOM). LOOM takes each protein in turn as a query protein, and carries out function prediction with the remaining proteins in the network. As the bootstrapping in *Gibbs sampling* based collective classification does not result in updating of the query protein, therefore we use the *majority* method to predict protein functions in LOOM cross validation. Then, the annotated protein proportion is changed from 10% to 90%, and the average performance of 10 experiments is reported for each of all proportions. The *majority* method is not suitable in this setting because it is a local neighbor counting approach and does not work well in sparsely-labeled network. Thus, the *Gibbs sampling* based collective classification is used to predict protein functions. The main hardware configuration of an Inter dual-core processor (3 GHz) and 16GB RAM, with a Linux operating system, and Python 3.0 is as the programming environment for running the algorithms.

Finally, as in Bogdanov and Singh (2010), the ratio of the number of *true positive* (TP) predictions to the number of *false positive*predictions (FP) is produced in the cross validation, i.e. TP/FP is used to assess prediction accuracy of PPI networks. We define the overall *i*th rank *true positive* (TP) as the number of proteins whose *i*th rank predicted function $c_{x}^{i}$ is one of the true functions of protein *V*_*x*_, and the overall *i*th rank *false positive* (FP) as the number of proteins whose *i*th rank predicted function $c_{x}^{i}$ is not one of the true functions of protein *V*_*x*_.

### 3.2 Similarity Index Selection and the Effect of the Parameters *k* and *λ*

In this study, in addition to sequence similarity, the PPI networks are reconstructed and enriched by using three local similarity indices (CN, Jaccard and FS)and two global similarity indices (Katz and RWR). In order to choose the best ones for the following experiments, the performance differences between the five similarity indices are evaluated over the two datasets of A and B. The experimental results over the dataset A are presented in Supplementary Table S3 and Table 1, and ones over the Dataset B listed in Table 2. Using FS as the local similarity index and RWR as the global similarity index generally achieve the best performance. Hence, FS and RWR are selected as the local similarity index and global similarity index, respectively in the following experiments.

**TABLE 1:** Comparison of performance differences between similarity indices (Dataset A: *M.musculus*).

Indices	Molecular function			Biological process				Cellular component		
	1st rank	2nd rank	3rd rank	1st rank	2nd rank	3rd rank	4th rank	1st rank	2nd rank	3rd rank
Origin	0.28	0.12	0.10	0.39	0.23	0.13	0.09	1.63	0.45	0.24
CN	0.21	0.09	0.07	0.27	0.22	0.14	0.09	1.44	0.47	0.16
Jaccard	0.30	**0.16**	0.11	**0.49**	**0.30**	0.129	0.11	1.94	0.56	0.25
FS	**0.33**	0.15	**0.15**	0.47	0.28	**0.16**	**0.12**	**2.13**	**0.61**	**0.27**
CN+	0.27	0.14	0.10	0.37	0.26	0.14	0.11	1.70	0.54	0.21
Jaccard+	0.35	0.16	0.12	**0.54**	**0.34**	0.15	0.12	2.03	0.62	0.27
FS+	**0.38**	**0.16**	**0.15**	0.52	0.30	**0.16**	**0.14**	**2.23**	**0.69**	**0.29**
Katz	0.29	0.13	0.12	0.45	0.23	**0.17**	0.11	1.70	0.54	0.28
RWR	**0.32**	**0.15**	**0.13**	**0.49**	**0.26**	0.16	**0.12**	**2.23**	**0.61**	**0.30**
Katz+	0.31	**0.16**	0.14	0.47	0.26	**0.19**	**0.14**	2.13	0.59	0.27
RWR+	**0.35**	0.15	**0.16**	**0.52**	**0.28**	0.17	0.12	**2.45**	**0.64**	**0.33**

**TABLE 2:** Comparison of performance differences between similarity indices (Dataset B).

Indices	*S.cerevisiae*			*M.musculus*		
	1st rank	2nd rank	3rd rank	1st rank	2nd rank	3rd rank
Origin	2.23	0.75	0.49	1.94	1.49	0.82
CN	1.50	0.54	0.29	1.28	0.69	0.43
Jaccard	1.55	0.62	0.39	1.51	1.13	**0.79**
FS	**1.70**	**0.64**	**0.41**	**1.56**	**1.22**	0.75
CN+	1.85	0.67	0.43	1.63	1.27	0.72
Jaccard+	1.95	0.65	**0.49**	1.78	1.33	0.78
FS+	**2.13**	**0.72**	0.47	**1.92**	**1.51**	**0.81**
Katz	1.63	0.62	0.41	1.70	**1.33**	0.75
RWR	**1.78**	**0.67**	**0.43**	**1.78**	1.27	**0.79**
Katz+	1.86	0.64	0.47	1.86	**1.51**	0.79
RWR+	**2.23**	**0.75**	**0.52**	**2.03**	1.49	**0.85**

The effect of two parameters on the performance of network reconstruction and edge enrichment are also examined in our study. The first one is the number of similarity-inferred edges *k*. The prediction performance on the Datasets of A and B is listed in Supplementary Table S4, Table 3, and Table 4, with the varying values of *k*. For both the datasets A and B, experimental results show that BLAST roughly achieves the best performance by setting *k* = 5. When the values of *k* = {10, 30, 50, 100} are used for FS and RWR, using *k* = 30 or *k* = 50 generally works best in most cases, and the overall performance is relatively robust for the reconstructed or enriched networks. Hence, in the following experiments, the parameter value of *k* is used as 5, 30, 30 for BLAST, FS and RWR, respectively.

**TABLE 3:** The influence of the parameter of *k* (*M.musculus* in Dataset A).

Indices	Molecular function			Biological process				Cellular component		
	1st rank	2nd rank	3rd rank	1st rank	2nd rank	3rd rank	4th rank	1st rank	2nd rank	3rd rank
Origin	0.28	0.12	0.10	0.39	0.23	0.13	0.09	1.63	0.45	0.24
BLAST 1	0.34	0.19	0.09	0.30	0.16	0.08	0.04	0.79	0.34	0.13
BLAST 5	0.43	0.26	0.13	**0.45**	**0.22**	0.12	0.08	**0.98**	**0.38**	0.18
BLAST 10	**0.45**	**0.27**	0.11	0.43	0.18	**0.13**	0.09	0.96	0.35	0.17
BLAST 15	0.41	0.21	**0.13**	0.42	0.20	0.11	**0.09**	0.92	0.33	**0.19**
BLAST+1	0.39	0.24	0.15	0.47	0.26	0.15	0.12	1.71	0.49	0.27
BLAST+5	0.47	**0.29**	**0.23**	**0.56**	0.30	**0.18**	**0.14**	**2.02**	**0.67**	0.32
BLAST+10	**0.49**	0.24	0.21	0.54	0.32	0.14	0.11	1.94	0.58	0.29
BLAST+15	0.46	0.27	0.20	0.49	**0.34**	0.15	0.12	1.86	0.62	**0.33**
FS 10	0.30	0.14	0.12	0.42	0.24	0.14	0.09	1.71	0.54	0.23
FS 30	0.33	0.15	**0.15**	**0.47**	0.28	0.16	0.12	**2.13**	0.61	0.27
FS 50	**0.35**	0.16	0.17	0.46	**0.30**	**0.18**	0.14	2.04	**0.64**	**0.28**
FS 100	0.32	**0.18**	0.12	0.45	0.27	0.17	**0.15**	1.95	0.57	0.26
FS+10	0.32	0.14	0.12	0.26	0.14	0.14	0.10	1.95	0.58	0.25
FS+30	0.39	0.16	0.15	0.52	**0.30**	**0.16**	0.14	**2.23**	**0.70**	**0.30**
FS+50	**0.41**	0.15	0.11	**0.54**	0.24	0.14	**0.14**	2.21	0.67	0.25
FS+100	0.38	**0.18**	**0.16**	0.50	0.27	0.16	0.13	2.07	0.64	0.27
RWR 10	0.25	0.13	0.11	0.41	0.21	0.14	0.09	1.86	0.54	0.24
RWR 30	**0.32**	0.15	0.13	**0.49**	**0.26**	0.16	0.11	2.23	**0.61**	**0.30**
RWR 50	0.31	**0.16**	0.11	0.47	0.21	**0.17**	0.12	**2.33**	0.58	0.27
RWR 100	0.29	0.15	**0.15**	0.44	0.22	0.16	**0.14**	2.12	0.55	0.30
RWR+10	0.29	0.14	0.13	0.47	0.23	0.15	0.12	2.13	0.57	0.29
RWR+30	**0.35**	0.15	**0.16**	**0.52**	**0.28**	**0.18**	0.12	**2.45**	**0.64**	0.33
RWR+50	0.34	**0.16**	0.15	0.49	0.28	0.14	**0.13**	2.36	0.62	0.32
RWR+100	0.31	0.15	0.15	0.46	0.25	0.16	0.12	2.23	0.58	**0.36**

**TABLE 4:** The effect of the parameter *k* (Dataset B).

Indices	*S.cerevisiae*			*M.musculus*		
	1st rank	2nd rank	3rd rank	1st rank	2nd rank	3rd rank
Origin	2.23	0.75	0.49	1.94	1.49	0.82
BLAST 1	0.96	0.37	0.17	1.28	0.59	0.35
BLAST 5	1.18	**0.43**	**0.28**	1.63	**0.75**	0.45
BLAST 10	**1.21**	0.39	0.24	**1.70**	0.72	0.41
BLAST 15	1.15	0.42	0.26	1.56	0.70	**0.50**
BLAST+1	2.11	0.64	0.45	2.15	1.51	0.82
BLAST+5	**2.83**	**0.82**	0.64	**2.45**	**1.63**	**0.87**
BLAST+10	2.57	0.75	**0.65**	2.33	1.57	0.85
BLAST+15	2.40	0.69	0.62	2.28	1.49	0.76
FS 10	1.53	0.55	0.38	1.33	1.06	0.68
FS 30	1.72	**0.64**	**0.41**	1.56	**1.22**	0.75
FS 50	**1.75**	0.57	0.38	1.64	1.19	**0.79**
FS 100	1.63	0.61	0.37	**1.68**	1.18	0.73
FS+10	1.93	0.65	0.40	1.85	0.42	0.79
FS+30	**2.13**	**0.72**	0.47	**1.92**	**1.51**	**0.81**
FS+50	2.05	0.70	0.44	1.83	1.40	0.76
FS+100	1.90	0.67	**0.49**	1.92	1.45	0.78
RWR 10	1.50	0.57	0.36	1.57	1.08	0.69
RWR 30	**1.78**	**0.67**	0.43	**1.78**	1.27	0.79
RWR 50	1.72	0.63	0.40	1.69	**1.31**	0.74
RWR 100	1.70	0.61	**0.45**	1.64	1.17	**0.82**
RWR+10	2.00	0.70	0.46	1.88	1.40	0.69
RWR+30	**2.23**	0.75	**0.52**	**2.03**	**1.49**	**0.85**
RWR+50	2.11	0.72	0.49	1.94	1.43	0.82
RWR+100	1.94	**0.81**	0.48	1.82	1.45	0.75

The second parameter *λ* dominates the tradeoff between explicit edges and similarity-inferred edges. Further, the effect of the parameter *λ* is evaluated on the prediction performance when it varies from 0.1 to 0.9. The results on the Dataset A are listed in Supplementary material (see Supplementary Table S5) and Table 5, and ones on the Dataset B in Table 6, respectively. Generally, the *λ* value has a relatively small impact on prediction accuracy, unless it is too large or too small. In the following experiments, the *λ* value is set uniformly at 0.7.

**TABLE 5:** The influence of the parameter *λ* (*M.musculus* in Dataset A).

Indices	Molecular function			Biological process				Cellular component		
	1st rank	2nd rank	3rd rank	1st rank	2nd rank	3rd rank	4th rank	1st rank	2nd rank	3rd rank
Origin	0.28	0.12	0.10	0.39	0.23	0.13	0.09	1.63	0.45	0.24
BLAST+0.1	0.38	0.21	0.11	0.43	0.24	0.12	0.13	1.52	0.41	0.20
BLAST+0.3	0.40	0.25	0.18	0.49	0.29	**0.17**	0.11	1.65	0.54	0.25
BLAST+0.5	0.44	**0.30**	0.16	0.537	0.27	0.15	**0.15**	1.85	0.62	0.28
BLAST+0.7	**0.47**	0.29	**0.23**	**0.56**	**0.32**	0.16	0.14	**2.02**	**0.67**	**0.34**
BLAST+0.9	0.33	0.16	0.15	0.42	0.23	0.13	0.10	1.76	0.55	0.27
FS+0.1	0.31	0.14	0.12	0.49	0.24	0.15	0.13	1.94	0.59	0.27
FS+0.3	0.35	0.16	**0.16**	**0.53**	**0.33**	0.13	0.10	1.86	0.68	0.25
FS+0.5	0.37	0.15	0.13	0.49	0.31	**0.18**	0.11	2.04	0.63	0.28
FS+0.7	**0.39**	**0.16**	0.15	0.52	0.30	0.16	**0.14**	**2.23**	**0.70**	**0.30**
FS+0.9	0.30	0.13	0.10	0.42	0.222	0.14	0.11	1.86	0.57	0.26
RWR+0.1	0.30	0.13	0.12	0.47	0.29	0.16	0.12	2.12	0.59	0.28
RWR+0.3	0.33	0.15	0.14	0.50	**0.31**	0.11	0.08	2.22	0.64	0.32
RWR+0.5	0.35	0.13	**0.17**	0.50	0.24	0.16	0.10	2.32	**0.74**	0.30
RWR+0.7	**0.37**	**0.17**	0.14	**0.52**	0.28	**0.18**	**0.12**	**2.45**	0.64	**0.33**
RWR+0.9	0.30	0.13	0.10	0.43	0.26	0.14	0.10	1.94	0.57	0.27

**TABLE 6:** The influence of the parameter *λ* (Dataset B).

Indices	*S.cerevisiae*			*M.musculus*		
	1st rank	2nd rank	3rd rank	1st rank	2nd rank	3rd rank
Origin	2.23	0.75	0.49	1.94	1.49	0.82
BLAST+0.1	1.56	0.63	0.41	1.76	1.28	0.72
BLAST+0.3	1.89	0.70	0.58	2.01	1.44	0.78
BLAST+0.5	2.56	0.75	**0.66**	2.34	1.37	0.82
BLAST+0.7	**2.83**	**0.82**	0.64	**2.45**	**1.63**	**0.87**
BLAST+0.9	2.36	0.79	0.56	2.12	1.51	0.85
FS+0.1	1.86	0.66	0.42	1.70	1.33	0.74
FS+0.3	1.93	0.64	0.45	1.86	1.38	0.81
FS+0.5	2.06	0.69	0.43	**2.02**	1.44	**0.87**
FS+0.7	**2.13**	0.72	**0.47**	1.92	1.51	0.84
FS+0.9	1.99	**0.75**	0.41	1.88	**1.62**	0.79
RWR+0.1	1.82	0.62	0.42	1.65	1.38	0.77
RWR+0.3	1.94	0.65	0.48	1.83	1.44	**0.83**
RWR+0.5	2.02	0.71	**0.54**	1.95	1.46	0.73
RWR+0.7	**2.23**	**0.75**	0.52	**2.03**	**1.49**	0.82
RWR+0.9	2.12	0.69	0.47	1.92	1.43	0.77

### 3.3 Performance Evaluation on Dataset A

The performance comparison of reconstructed and enriched networks with that of the original networks is first carried out by leave-one-out validation. The top protein function prediction is selected according to the average number of useful functions per protein in the PPI networks. Therefore, only the top 2 predictions are performed on the PPI networks of *S.cerevisiae* in the Dataset A, and the top 3 or 4 predictions are examined for *M.musculus* in Dataset A.

**FIGURE 1:**
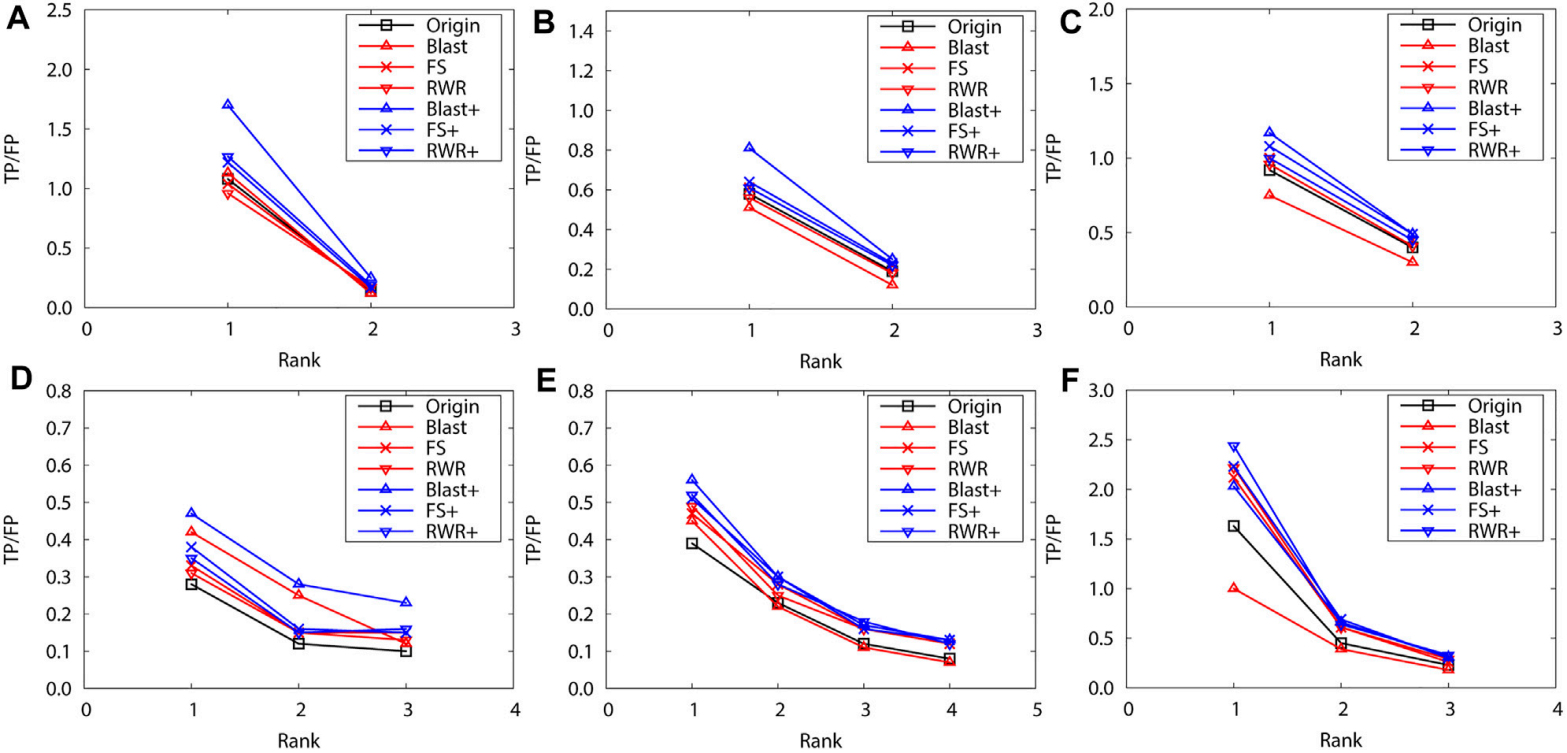
The performance evaluation by leave-one-out validation over the PPI networks (Dataset A: *S.cerevisiae* and *M.musculus*). Here, the sub figures in the horizontal and vertical directions represent the experimental results for the PPI networks of different data sets and function types, respectively. Horizontally, the top three subplots represent ones on *S.cerevisiae*, and the bottom for ones on *M.musculus*. **(A)** and **(D)** Molecular function, **(B)** and **(E)** Biological process, **(C)** and **(F)** Cellular component.

Obviously, edge enrichment gains more accurate predictions than network reconstruction and original networks, due to the combination of explicit and implicit (similarity-inferred) edges (Figure 1). The results clearly indicate that edge enrichment indeed gains better prediction performance by adding similarity-inferred edges to PPI networks. BLAST-enriched networks always worke best, while BLAST-reconstructed networks always work worst. This is because BLAST-inferred edges are based on protein sequence information that is short in the original networks. The useful information in the original network greatly increases by adding BLAST-inferred edges, and consequently boosts prediction accuracy. However, in the reconstructed networks, the original PPI edges are put aside first, BLAST-reconstructed networks contain only protein sequence information, and thus performe worst. The experimental results also validate that FS-reconstructed networks and RWR-reconstructed networks work better than the original networks in most cases. This is because the reconstructed networks filter out noisy or spurious interactions in the original PPI networks.

**FIGURE 2:**
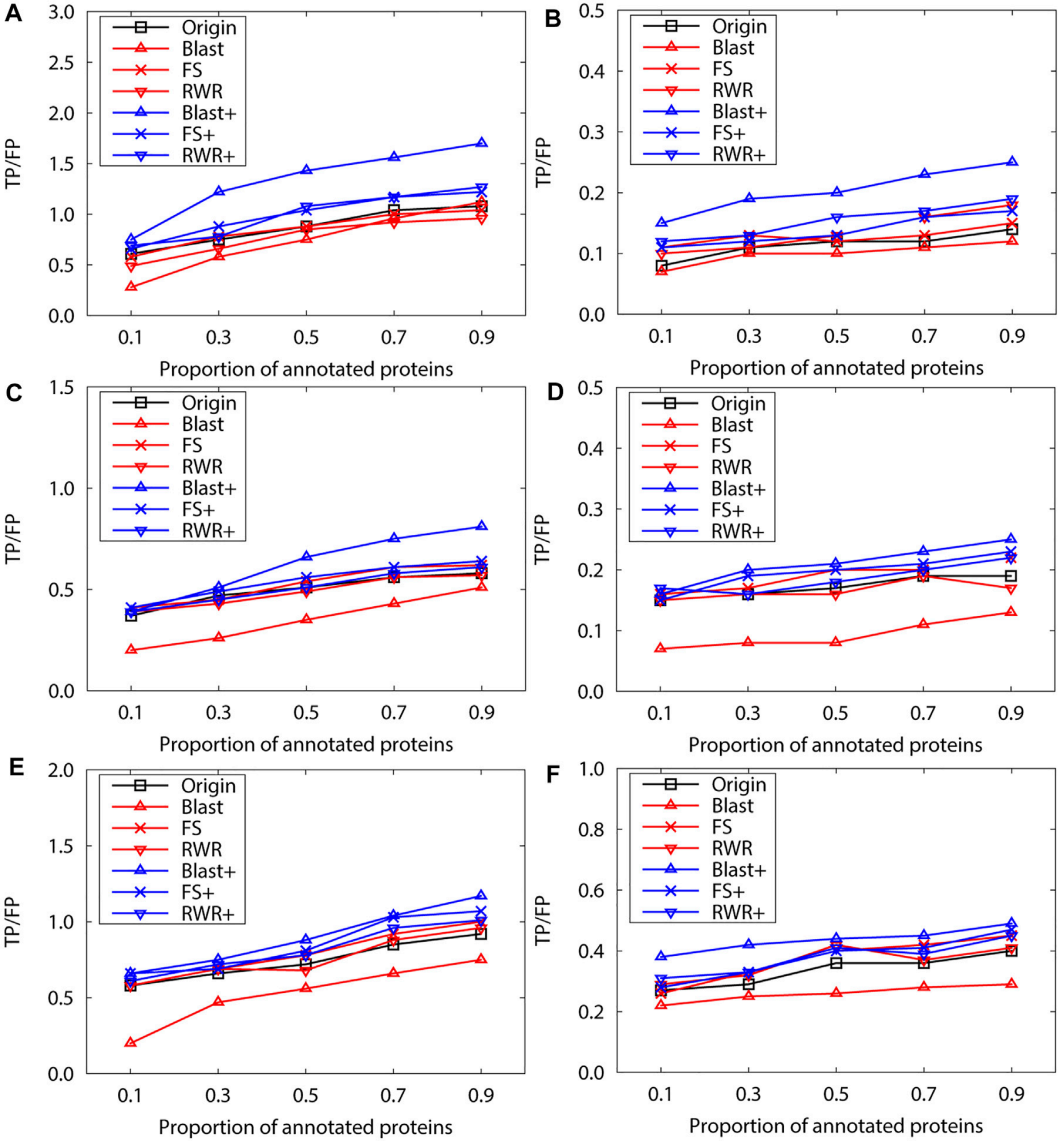
The performance evaluation over the sparsely-labeled networks (Dataset A: *S.cerevisiae*). Here, the sub figures in the horizontal and vertical directions represent the experimental results for the PPI networks of different function types and rank predicted functions, respectively. Horizontally, the top two subplots represent ones of molecular function, the middle for ones of biological process, and the bottom for ones of cellular component **(A)**, **(C)** and **(E)** first rank predicted function, **(B)**, **(D)** and **(F)** second rank predicted function.

**FIGURE 3:**
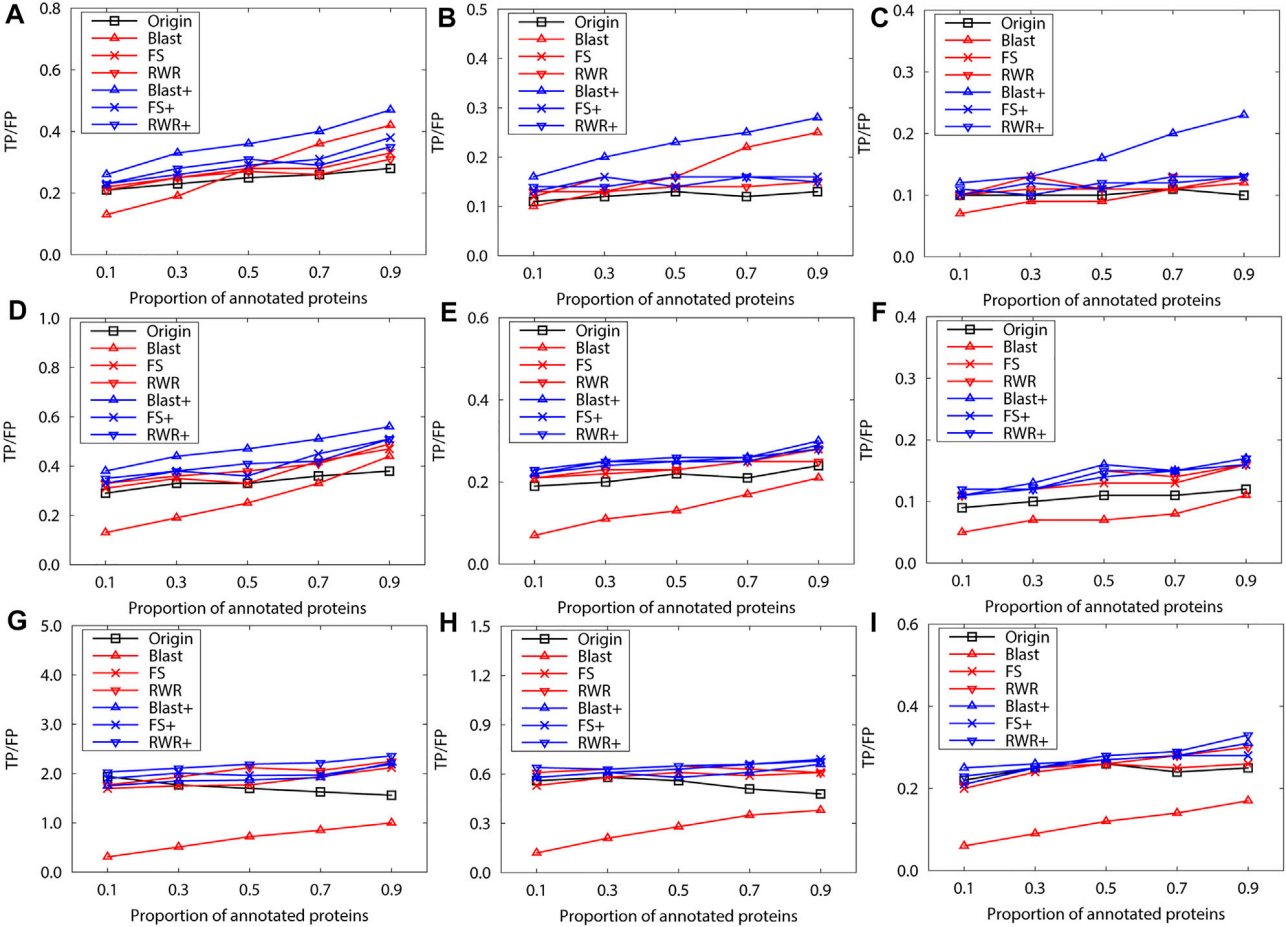
The performance evaluation over the sparsely-labeled networks (Dataset A: *M.musculus*). Here, the sub figures in the horizontal and vertical directions represent the experimental results for the PPI networks of different function types and rank predicted functions, respectively. Horizontally, the top three subplots represent ones on the PPI networks of molecular function, the middle for ones of biological process, and the bottom for ones of cellular component **(A)**, **(D)** and **(G)** first rank predicted function, **(B)**, **(E)** and **(H)** second rank predicted function, **(C)**, **(F)** and **(I)** third rank predicted function.

We further evaluate prediction accuracy of these three kinds of networks by using Gibbs Sampling in sparse-labeled PPI networks. Concretely, in PPI networks, the annotated protein proportion is changed from 0.1 to 0.9, and the remaining protein functions are predicted. For each proportion of the annotated proteins, the average prediction accuracy of running 10 experiments is presented on the PPI networks of *S.cerevisiae* (Figure 2)and *M.musculus* (Figure 3), respectively. The enrichment gains more accurate predictions than network reconstruction and original networks. The BLAST-enriched networks always work the best, while the BLAST-reconstructed networks always perform the worst. As expected, the experimental results also validate that FS-reconstructed networks and RWR-reconstructed networks generally performe better than the original networks. As the annotated protein proportion in the original networks increases, the prediction performance gets better for most networks, especially for the 1-st rank function. However, the prediction performance of the original network slightly declines as its annotated protein proportion increases (Figure 3G, H).

### 3.4 Performance Evaluation on Dataset B

**FIGURE 4:**
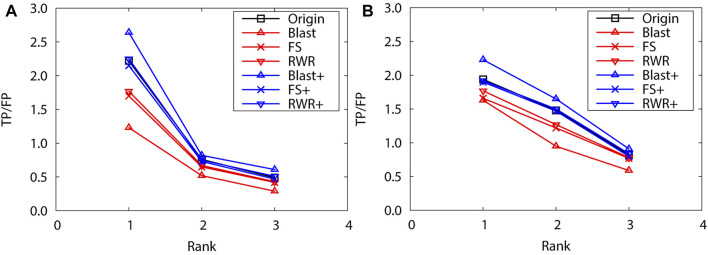
The performance evaluation by leave-one-out validation over the PPI networks (Dataset B: *S.cerevisiae* and *M.musculus*) **(A)** *S.cerevisiae* **(B)** *M.musculus*.

As above, the performance of reconstructed and enriched networks is first compared with that of the original networks by leave-one-out validation. Here, the top 3 protein function predictions are considered for both PPI networks of *S. cerevisiae* and *M. musculus*. As expected, edge enrichment gaines higher accurate predictions than network reconstruction and original networks. Moreover, BLAST-enriched networks get best, while the BLAST-reconstructed networks always work worst (Figure 4). The reasons are the same as for the dataset A.

**FIGURE 5:**
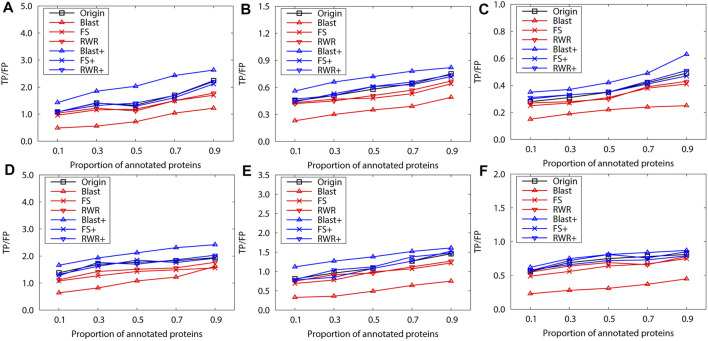
The performance evaluation over the sparsely-labeled networks (Dataset B: *S.cerevisiae* and *M.musculus*). Here, the sub figures in the horizontal and vertical directions represent the experimental results for different data types and rank predicted functions, respectively. Horizontally, the top three subplots represent ones over the dataset of *S.cerevisiae*, and the bottom for ones of *M.musculus*. **(A)** and **(D)** first rank predicted function, **(B)** and **(E)** second rank predicted function, **(C)** and **(F)** third rank predicted function.

Next, we evaluate the prediction performance of these networks in sparse-labeled conditions with the collective classification method. Similarly, the average prediction performance is generated over running 10 experiments, with the annotated-protein proportion varying from 0.1 to 0.9. Generally, the experimental results present a similar trend to the above for the dataset A (Figure 5). However, FS-reconstructed networks and RWR-reconstructed networks do not outperform the original networks, due to the quality properties of the dataset itself. This is mainly because many informative interactions are deleted and the prediction performance is impaired when reconstructing the networks based on similarity.

To validate this point, 10% and 50% interactions of the original network of the dataset B are randomly selected to construct two sparse networks. The leave-one-out validation is then performed over the two sparse networks. The selection process have two steps: First, a random weight is assigned to each edge of the original network, and a minimum spanning tree is constructed on the new network. The randomness of the minimum spanning tree (*MST*) is ensured by the random weights, and *MST* ensures the connectivity of the sparse network. Second, the *MST* is expanded by adding a number of edges, which are randomly selected from the original network (but not already on the *MST*). Hence, the number of edges in the sparse network is equal to 10% or 50% of edges in the original network. The sparse network preserves the basic topological properties of the original network.

**FIGURE 6:**
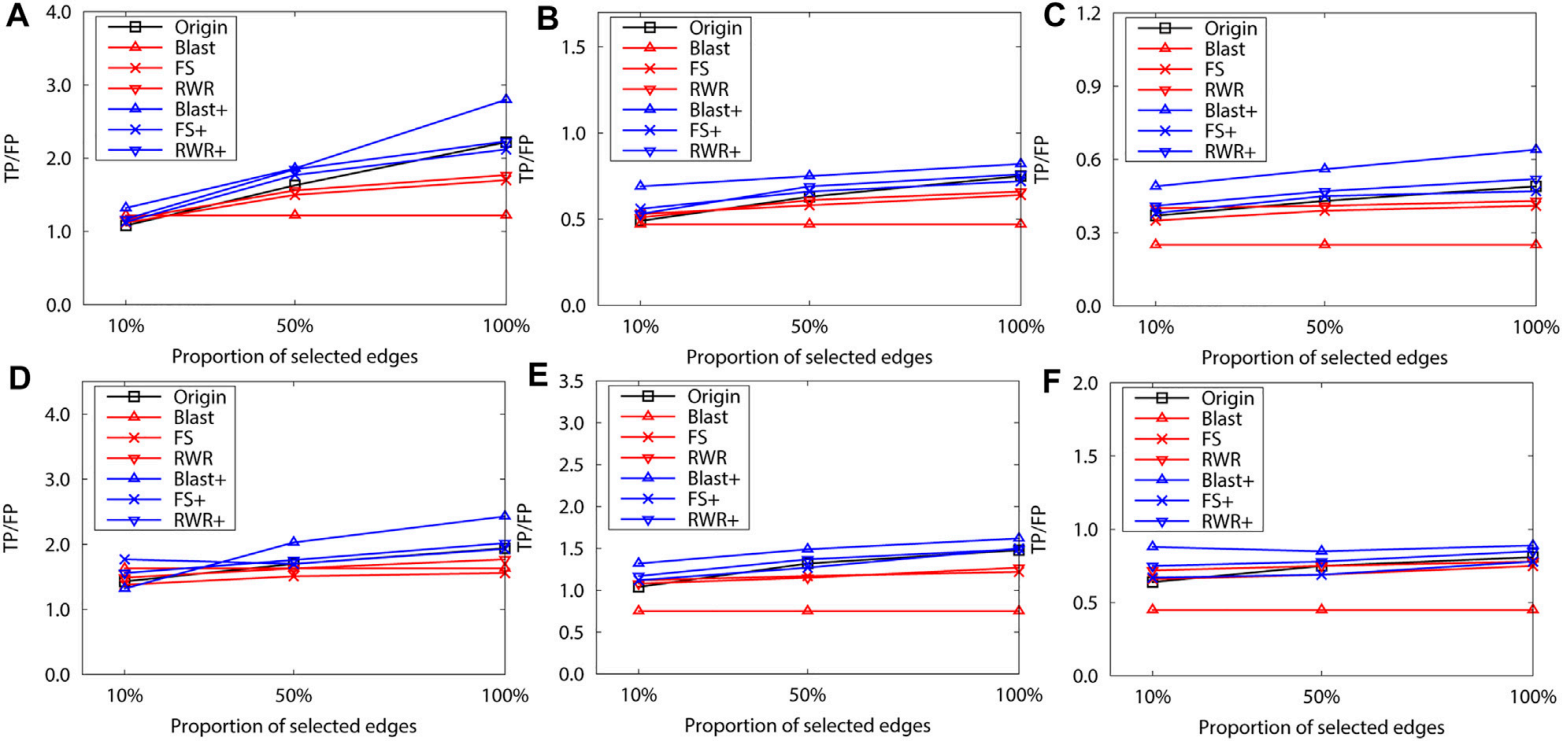
The performance evaluation by leave-one-out validation over the PPI networks (Dataset B: *S.cerevisiae* and *M.musculus*). Here, the sub figures in the horizontal and vertical directions represent the experimental results for different data types and rank predicted functions, respectively. Horizontally, the top three subplots represent ones over the dataset of *S.cerevisiae*, and the bottom for ones of *M.musculus* **(A)** and **(D)** first rank predicted function, **(B)** and **(E)** second rank predicted function, **(C)** and **(F)** third rank predicted function.

The final experimental results also confirm the above-mentioned phenomenon. For example, in Figure 6, the FS-reconstructed networks and the RWR-reconstructed networks work better than the original networks when the networks are very sparse (e.g. 10%). However, as the networks become denser, the FS-reconstructed networks and the RWR-reconstructed networks get worse than the original networks.

## 4 Conclusion

The systematic comparison of two network transformation approaches (network reconstruction and edge enrichment) is performed using three different protein similarity metrics (sequence similarity, local and global similarity). In summary, edge enrichment performs better than network reconstruction and original networks, while network reconstruction is more effective on relatively small and incomplete PPI networks. The edge enrichment of PPI networks based on sequence similarity outperforms those based on both local and global similarity. As the PPI networks become more and more complete, the effectiveness of both edge enrichment and network reconstruction will decrease or relatively decrease.

Research efforts will be further expanded in future, which include: 1) how the removal of noisy edges and addition of informative edges affect the prediction performance; 2) a combining approach that combines the best properties of all these indices is developed since the similarity indices considered here have different properties and performances.

## Data Availability

Publicly available datasets were analyzed in this study. This data can be found here: (1) Datasets A: BioGRID, https://downloads.thebiogrid.org/BioGRID. (2) Datasets A: STRING, https://string-db.org.

## References

[B1] Abdollahi S. Lin P.-C. Chiang J.-H. (2021). Winbinvec: Cancer-Associated Protein-Protein Interaction Extraction and Identification of 20 Various Cancer Types and Metastasis Using Different Deep Learning Models. IEEE J. Biomed. Health Inform. 25, 4052–4063. 10.1109/JBHI.2021.3093441 34185653

[B2] Adamcsek B. Palla G. Farkas I. J. Derényi I. Vicsek T. (2006). CFinder: Locating Cliques and Overlapping Modules in Biological Networks. Bioinformatics 22, 1021–1023. 10.1093/bioinformatics/btl039 16473872

[B3] Altschul S. Madden T. Schäffer A. A. Zhang J. Zhang Z. Miller W. (1997). Gapped BLAST and PSI-BLAST: a New Generation of Protein Database Search Programs. Nucleic Acids Res. 25, 3389–3402. 10.1093/nar/25.17.3389 9254694 PMC146917

[B4] Ashburner M. Ball C. A. Blake J. A. Botstein D. Butler H. Cherry J. M. (2000). Gene Ontology: Tool for the Unification of Biology. Nat. Genet. 25, 25–29. 10.1038/75556 10802651 PMC3037419

[B5] Barrell D. Dimmer E. Huntley R. P. Binns D. O'Donovan C. Apweiler R. (2009). The GOA Database in 2009--an Integrated Gene Ontology Annotation Resource. Nucleic Acids Res. 37, D396–D403. 10.1093/nar/gkn803 18957448 PMC2686469

[B6] Berggård T. Linse S. James P. (2007). Methods for the Detection and Analysis of Protein-Protein Interactions. Proteomics 7, 2833–2842. 10.1002/pmic.200700131 17640003

[B7] Bogdanov P. Singh A. K. (2010). Molecular Function Prediction Using Neighborhood Features. Ieee/acm Trans. Comput. Biol. Bioinf. 7, 208–217. 10.1109/TCBB.2009.81 20431141

[B8] Chen Y. Wang W. Liu J. Feng J. Gong X. (2020). Protein Interface Complementarity and Gene Duplication Improve Link Prediction of Protein-Protein Interaction Network. Front. Genet. 11, 291. 10.3389/fgene.2020.00291 32300358 PMC7142252

[B9] Chua H. N. Sung W.-K. Wong L. (2007). An Efficient Strategy for Extensive Integration of Diverse Biological Data for Protein Function Prediction. Bioinformatics 23, 3364–3373. 10.1093/bioinformatics/btm520 18048396

[B10] Chua H. N. Sung W.-K. Wong L. (2006). Exploiting Indirect Neighbours and Topological Weight to Predict Protein Function from Protein-Protein Interactions. Bioinformatics 22, 1623–1630. 10.1093/bioinformatics/btl145 16632496

[B11] Dunn R. Dudbridge F. Sanderson C. M. (2005). The Use of Edge-Betweenness Clustering to Investigate Biological Function in Protein Interaction Networks. BMC Bioinformatics 6, 39. 10.1186/1471-2105-6-39 15740614 PMC555937

[B12] Hu L. Huang T. Shi X. Lu W.-C. Cai Y.-D. Chou K.-C. (2011). Predicting Functions of Proteins in Mouse Based on Weighted Protein-Protein Interaction Network and Protein Hybrid Properties. PLOS ONE 6, e14556. 10.1371/journal.pone.0014556 21283518 PMC3023709

[B13] Kuchaiev O. Milenković T. Memišević V. Hayes W. Pržulj N. (2010). Topological Network Alignment Uncovers Biological Function and Phylogeny. J. R. Soc. Interf. 7, 1341–1354. 10.1098/rsif.2010.0063 PMC2894889 20236959

[B14] Liben-Nowell D. Kleinberg J. (2007). The Link-Prediction Problem for Social Networks. J. Am. Soc. Inf. Sci. 58, 1019–1031. 10.1002/asi.20591

[B15] Lü L. Zhou T. (2011). Link Prediction in Complex Networks: A Survey. Physica A: Stat. Mech. its Appl. 390, 1150–1170. 10.1016/j.physa.2010.11.027

[B16] Newman M. E. J. (2001). Clustering and Preferential Attachment in Growing Networks. Phys. Rev. E 64, 025102. 10.1103/PhysRevE.64.025102 11497639

[B17] Ofran Y. Punta M. Schneider R. Rost B. (2005). Beyond Annotation Transfer by Homology: Novel Protein-Function Prediction Methods to Assist Drug Discovery. Drug Discov. Today 10, 1475–1482. 10.1016/S1359-6446(05)03621-4 16243268

[B18] Patel S. Tripathi R. Kumari V. Varadwaj P. (2017). Deepinteract: Deep Neural Network Based Protein-Protein Interaction Prediction Tool. Cbio 12, 551–557. 10.2174/1574893611666160815150746

[B19] Schwikowski B. Uetz P. Fields S. (2000). A Network of Protein-Protein Interactions in Yeast. Nat. Biotechnol. 18, 1257–1261. 10.1038/82360 11101803

[B20] Sen P. Namata G. Bilgic M. Getoor L. Galligher B. Eliassi-Rad T. (2008). Collective Classification in Network Data. AIMag 29, 93–106. 10.1609/aimag.v29i3.2157

[B21] Sharan R. Ulitsky I. Shamir R. (2007). Network‐based Prediction of Protein Function. Mol. Syst. Biol. 3, 88. 10.1038/msb4100129 17353930 PMC1847944

[B22] Singh R. Xu J. Berger B. (2008). Global Alignment of Multiple Protein Interaction Networks with Application to Functional Orthology Detection. Proc. Natl. Acad. Sci. 105, 12763–12768. 10.1073/pnas.0806627105 18725631 PMC2522262

[B23] Sleator R. D. Walsh P. (2010). An Overview of In Silico Protein Function Prediction. Arch. Microbiol. 192, 151–155. 10.1007/s00203-010-0549-9 20127480

[B24] Solava R. W. Michaels R. P. Milenković T. (2012). Graphlet-based Edge Clustering Reveals Pathogen-Interacting Proteins. Bioinformatics 28, i480–i486. 10.1093/bioinformatics/bts376 22962470 PMC3436803

[B25] Stark C. Breitkreutz B.-J. Chatr-Aryamontri A. Boucher L. Oughtred R. Livstone M. S. (2011). The Biogrid Interaction Database: 2011 Update. Nucleic Acids Res. 39, D698–D704. 10.1093/nar/gkq1116 21071413 PMC3013707

[B26] Szklarczyk D. Franceschini A. Kuhn M. Simonovic M. Roth A. Minguez P. (2011). The String Database in 2011: Functional Interaction Networks of Proteins, Globally Integrated and Scored. Nucleic Acids Res. 39, D561–D568. 10.1093/nar/gkq973 21045058 PMC3013807

[B27] Täubig H. Buchner A. Griebsch J. (2006). Past: Fast Structure-Based Searching in the PDB. Nucleic Acids Res. 34, W20–W23. 10.1093/nar/gkl273 16844992 PMC1538836

[B28] Tong H. Faloutsos C. Pan J.-Y. (2008). Random Walk with Restart: Fast Solutions and Applications. Knowl Inf. Syst. 14, 327–346. 10.1007/s10115-007-0094-2

[B29] Waiho K. Afiqah‐Aleng N. Iryani M. T. M. Fazhan H. (2021). Protein-protein Interaction Network: an Emerging Tool for Understanding Fish Disease in Aquaculture. Rev. Aquacult. 13, 156–177. 10.1111/raq.12468

[B30] Wallace A. C. Laskowski R. A. Thornton J. M. (1996). Derivation of 3D Coordinate Templates for Searching Structural Databases: Application to Ser-His-Asp Catalytic Triads in the Serine Proteinases and Lipases. Protein Sci. 5, 1001–1013. 10.1002/pro.5560050603 8762132 PMC2143436

[B31] Wu Q. Ye Y. Ng M. K. Ho S.-S. Shi R. (2014). Collective Prediction of Protein Functions from Protein-Protein Interaction Networks. BMC Bioinformatics 15, S9. 10.1186/1471-2105-15-S2-S9 PMC4015526 24564855

[B32] Wu Z. Liao Q. Liu B. (2020). A Comprehensive Review and Evaluation of Computational Methods for Identifying Protein Complexes from Protein-Protein Interaction Networks. Brief. Bioinform. 21, 1531–1548. 10.1093/bib/bbz085 31631226

[B33] Xiong W. Liu H. Guan J. Zhou S. (2013). Protein Function Prediction by Collective Classification with Explicit and Implicit Edges in Protein-Protein Interaction Networks. BMC Bioinformatics 14, S4. 10.1186/1471-2105-14-S12-S4 PMC3848795 24267980

[B34] Xiong W. Xie L. Zhou S. Guan J. (2014). Active Learning for Protein Function Prediction in Protein-Protein Interaction Networks. Neurocomputing 145, 44–52. 10.1016/j.neucom.2014.05.075

[B35] Yang H. Wang M. Liu X. Zhao X.-M. Li A. (2021). PhosIDN: an Integrated Deep Neural Network for Improving Protein Phosphorylation Site Prediction by Combining Sequence and Protein-Protein Interaction Information. Bioinformatics, btab551. 10.1093/bioinformatics/btab551 34320631 PMC8665744

[B36] Ye Y. Godzik A. (2004). FATCAT: a Web Server for Flexible Structure Comparison and Structure Similarity Searching. Nucleic Acids Res. 32, W582–W585. 10.1093/nar/gkh430 15215455 PMC441568

[B37] Zhu H. Du X. Yao Y. (2020). Convsppis: Identifying Protein-Protein Interaction Sites by an Ensemble Convolutional Neural Network with Feature Graph. Cbio 15, 368–378. 10.2174/1574893614666191105155713

