# Supplementary material for: Protein Function Prediction Based on PPI Networks: Network Reconstruction vs Edge Enrichment
Source: Front Genet. 2021 Dec 14;12:758131. doi: 10.3389/fgene.2021.758131 (PMC8712557; doi:10.3389/fgene.2021.758131)
Supplement: Supplementary file 1 [file DataSheet1.pdf]

Supplementary Material

1 SUPPLEMENTARY TABLES AND FIGURES

Due to the limitation of article length, some experimental results are presented below. Table. S1 and Table. S2represent the statistical information of data sets A and B, respectively. Based on the same experimental data set (Dataset A: *S.cerevisiae*), Table. S3, Table. S4and Table. S5 indicate the performance differences between similarity indices, the effect of the number of similarity-inferred edges k and that of the parameter λ , respectively.

1.1 Tables

Table S1. Statistics of Dataset A.

Namespaces	*S.cerevisiae*		*M.musculus*	
	proteins	interactions	proteins	interactions
molecular function	1147	20013	715	1855
biological process	3277	54983	1046	2729
cellular component	4497	74422	1406	3614

Table S2. Statistics of Dataset B.

MIPS Functional Category		CYGD	MfunGD
01	METABOLISM	942	2662
02	ENERGY	151	603
04	STORAGE PROTEIN	0	0
10	CELL CYCLE AND DNA PROCESSING	1010	1113
11	TRANSCRIPTION	1078	2119
12	PROTEIN SYNTHESIS	480	490
14	PROTEIN FATE	1155	2484
16	PROTEIN WITH BINDING FUNCTION OR COFACTOR REQUIREMENT	1049	8369
18	REGULATION OF METABOLISM AND PROTEIN FUNCTION	249	1112
20	CELLULAR TRANSPORT, TRANSPORT FACILITIES AND TRANSPORT ROUTES	1042	2407
30	CELLULAR COMMUNICATION/SIGNAL TRANSDUCTION MECHANISM	234	4061
32	CELL RESCUE, DEFENSE AND VIRULENCE	554	769
34	INTERACTION WITH THE ENVIRONMENT	463	1486
36	SYSTEMIC INTERACTION WITH THE ENVIRONMENT	0	2073
38	TRANSPOSABLE ELEMENTS, VIRAL AND PLASMID PROTEINS	120	11
40	CELL FATE	273	1312
41	DEVELOPMENT (Systemic)	69	1042
42	BIOGENESIS OF CELLULAR COMPONENTS	863	979
43	CELL TYPE DIFFERENTIATION	452	370
45	TISSUE DIFFERENTIATION	0	426
47	ORGAN DIFFERENTIATION	0	559
70	SUBCELLULAR LOCALIZATION	0	9739
73	CELL TYPE LOCALIZATION	0	273
75	TISSUE LOCALIZATION	0	366
77	ORGAN LOCALIZATION	0	619

Table S3. Comparison of performance differences between similarity indices (Dataset A: *S.cerevisiae*).

Indices	molecular function		biological process		cellular component	
	1st	2nd	1st	2nd	1st	2nd
Origin	1.083	0.136	0.587	0.190	0.923	0.408
CN	0.493	0.163	0.370	0.136	0.528	0.266
Jaccard	0.885	**0.176**	0.493	0.163	0.982	0.408
FS	**1.041**	0.149	**0.613**	**0.220**	**1.003**	**0.449**
CN+	0.887	0.191	0.492	0.205	0.818	0.389
Jaccard+	1.043	**0.203**	0.563	0.187	**1.128**	0.429
FS+	**1.222**	0.176	**0.639**	**0.235**	1.086	**0.493**
Katz	0.923	0.169	0.471	**0.192**	0.881	0.351
RWR	**0.961**	**0.176**	**0.563**	0.176	**0.961**	**0.408**
Katz+	1.128	**0.193**	0.570	0.217	0.924	0.370
RWR+	**1.273**	0.190	**0.613**	**0.220**	**1.000**	**0.446**

Table S4. The effect of the number of similarity-inferred edges k (Dataset A: *S.cerevisiae*).

Indices	molecular function		biological process		cellular component	
	1st	2nd	1st	2nd	1st	2nd
Origin	1.083	0.136	0.587	0.190	0.923	0.408
BLAST 1	0.923	0.113	0.471	0.087	0.563	0.177
BLAST 5	1.128	0.123	**0.515**	0.120	**0.754**	**0.297**
BLAST 10	**1.147**	**0.138**	0.493	0.139	0.724	0.289
BLAST 15	1.040	0.122	0.447	**0.150**	0.696	0.282
BLAST+1	1.439	0.219	0.689	0.190	1.010	0.429
BLAST+5	**1.703**	0.250	**0.818**	**0.239**	1.174	**0.493**
BLAST+10	1.630	**0.265**	0.786	0.206	**1.225**	0.467
BLAST+15	1.615	0.239	0.754	0.224	1.130	0.449
FS 10	0.924	0.159	0.495	0.196	0.885	0.370
FS 30	**1.041**	0.146	**0.613**	**0.220**	1.003	**0.449**
FS 50	1.010	**0.161**	0.587	0.213	**1.040**	0.425
FS 100	0.963	0.152	0.563	0.207	0.981	0.429
FS+10	1.130	0.155	0.580	0.204	1.035	0.437
FS+30	1.222	**0.176**	**0.639**	**0.235**	**1.086**	**0.493**
FS+50	**1.243**	0.167	0.622	0.220	1.031	0.472
FS+100	1.235	0.161	0.610	0.232	1.042	0.440
RWR 10	0.639	0.126	0.374	0.116	0.657	0.356
RWR 30	**0.961**	0.176	**0.563**	**0.176**	**0.961**	**0.408**
RWR 50	0.923	**0.185**	0.519	0.139	0.875	0.380
RWR 100	0.864	0.173	0.470	0.151	0.827	0.357
RWR+10	1.033	0.145	0.538	0.176	0.852	0.415
RWR+30	**1.273**	0.190	**0.613**	**0.220**	**1.000**	0.446
RWR+50	1.222	**0.205**	0.587	0.192	0.967	0.420
RWR+100	1.150	0.176	0.552	0.216	0.910	**0.461**

Table S5. The effect of the parameter λ (Dataset A: *S.cerevisiae*).

Indices	molecular function		biological process		cellular component	
	1st	2nd	1st	2nd	1st	2nd
Origin	1.083	0.136	0.587	0.190	0.923	0.408
BLAST+0.1	1.273	0.178	0.617	0.195	0.873	0.378
BLAST+0.3	1.513	0.226	0.692	0.210	1.053	0.445
BLAST+0.5	1.630	**0.267**	0.781	0.223	1.133	0.407
BLAST+0.7	**1.703**	0.250	**0.818**	**0.239**	**1.174**	**0.493**
BLAST+0.9	1.381	0.197	0.648	0.201	0.983	0.429
FS+0.1	1.053	0.143	0.609	0.220	1.037	0.438
FS+0.3	1.133	0.161	0.641	0.239	1.093	0.417
FS+0.5	1.174	**0.196**	**0.673**	**0.261**	0.995	0.468
FS+0.7	**1.222**	0.176	0.639	0.235	**1.086**	**0.493**
FS+0.9	1.103	0.150	0.597	0.209	0.987	0.438
RWR+0.1	1.013	0.186	0.562	0.178	0.923	0.415
RWR+0.3	1.090	**0.210**	0.589	0.202	0.955	0.409
RWR+0.5	1.207	0.202	0.627	**0.245**	0.943	0.431
RWR+0.7	**1.273**	0.190	**0.673**	0.220	**1.000**	**0.446**
RWR+0.9	1.123	0.151	0.567	0.192	0.978	0.390
